# Minimal Invasive Percutaneous Fixation of Thoracic and Lumbar Spine Fractures

**DOI:** 10.1155/2012/141032

**Published:** 2012-07-16

**Authors:** Federico De Iure, Michele Cappuccio, Stefania Paderni, Giuseppe Bosco, Luca Amendola

**Affiliations:** Department of Orthopedics and Traumatology-Spine Surgery, Ospedale Maggiore “C.A. Pizzardi”, Largo Nigrisoli 2, 40100 Bologna, Italy

## Abstract

We studied 122 patients with 163 fractures of the thoracic and lumbar spine undergoing the surgical treatment by percutaneous transpedicular fixation and stabilization with minimally invasive technique. Patient followup ranged from 6 to 72 months (mean 38 months), and the patients were assessed by clinical and radiographic evaluation. The results show that percutaneous transpedicular fixation and stabilization with minimally invasive technique is an adequate and satisfactory procedure to be used in specific type of the thoracolumbar and lumbar spine fractures.

## 1. Introduction

Surgical treatment of thoracic and lumbar spine fractures is based on different factors. Type of fracture, neurological deficit, general conditions, and associated injuries affect both treatment and final result. Although type B and C fractures following AO-Magerl classification [1] require surgical treatment, most type A fractures without neurological involvement can be safely treated in a conservative way [2, 3]. Conservative treatment is a demanding procedure for the patient, and the risk of a final deformity has to be considered as a residual kyphosis can consistently worsen the quality of life of the patient. Moreover, some situations rule out the chance for a conservative treatment. In case of polytrauma, claustrophobia, psychological disease, venous disease or previous deep venous thrombosis, obesity, and bronchopulmonary diseases, conservative treatment is not advisable. Attention must also be paid to the fact that younger and active workers refuse the conservative treatment in order to avoid bed rest and an inactive period.

A traditional open surgery may be an overtreatment in all these cases, considering blood loss, possible complications, hospital stay, and delayed functional recovery. In this setting, a good option can be a percutaneous minimally invasive surgery (MIS) [4, 5]. This technique is suggested by the authors every time a conservative treatment is not indicated or advisable, and posterior open arthrodesis may represent an overtreatment.

## 2. Materials and Methods

From May 2005 to December 2011, 163 vertebral fractures of the thoracic and lumbar spine in 122 patients were stabilized. Eighty-tree patients were males and 39 females, the mean age was 48 years (from 15 to 85). Eighteen patients were polytrauma with an average Injury Severity Score of 25.2 (from 17 to 34). In those patient, percutaneous fixation was also intended to be a damage control procedure.

The most frequent location was the thoracolumbar junction (T12-L1). All fractures were classified according to the AO-Magerlclassification: the vast majority were type A fractures (A1 and A3), while type B or type C were recorded in a few cases (Table 1).

The most frequent construct was the monosegmental one (one level above and one below the fractured vertebra) in 96 cases. A multilevel construction was performed in 26 cases of multiple injuries. Overall, 553 pedicle screws were implanted with a percutaneous technique. 

In 18 cases, a bone substitute (cement and hydroxyapatite) was introduced in the fractured vertebra to fill the anterior gap left after reduction, to better support the anterior column.

**Figure 1:**
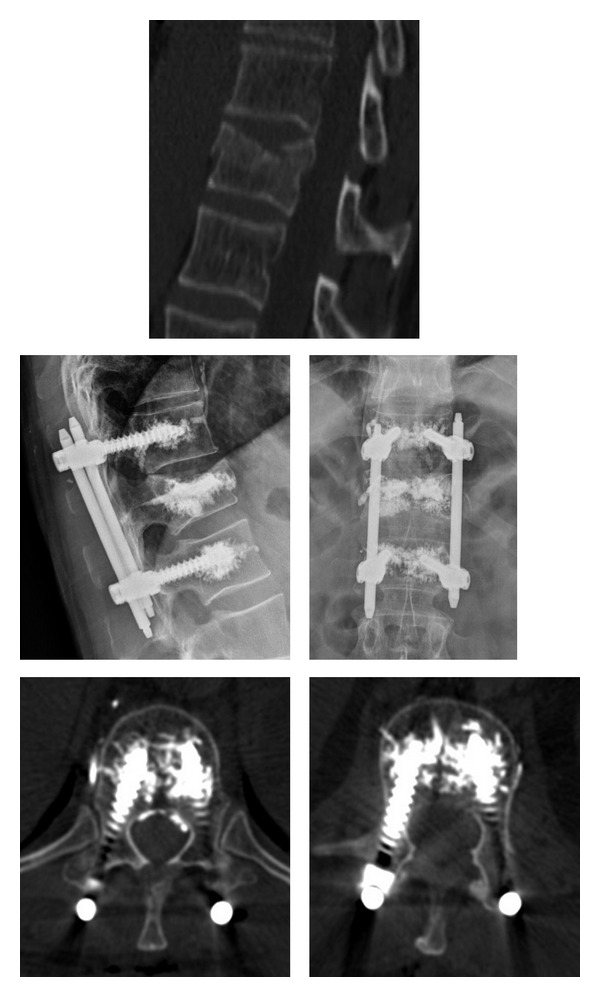
T12 type A3.1 fracture treated with cemented fenestrated screw and kyphoplasty.

In one of patients with poor bone stock due to osteoporosis, we used a fenestrated cemented screw, associated with kyphoplasty, to stabilize a T12 type A3 fracture (Figure 1).

In one case, the fracture stabilization was associated with a minimally invasive endoscopic-assisted discectomy and interbody fusion for a preexisting symptomatic degenerative disc-disease at the same level.

**Figure 2:**
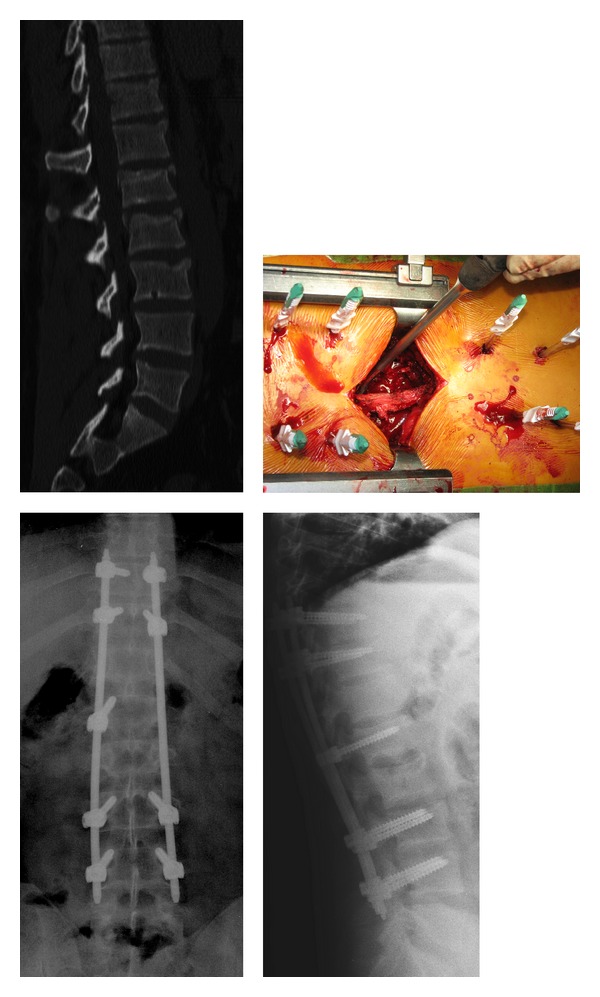
T11, T12, and L3 type A fractures associated with L1 and L2 type B fractures. Percutaneous stabilization from T10 to L4 and L1-L2 arthrodesis with a miniopen approach.

In another case where T11, T12, and L3 type A fractures were associated with L1 and L2 type B fractures, we performed a percutaneous stabilization from T10 to L4 and an L1-L2 arthrodesis with a miniopen approach (Figure 2).

In no other case fusion was associated to the MIS.

To monotrauma patients with type A1, A2, and A3.1 fractures without significant stenosis of the spinal canal, a conservative option consisting of cast and bed rest was also offered but was rejected in 85% of cases. In all cases, the impairment of the spinal canal was less than 30%, and local kyphosis was less than 20° except in one case. 

All patients underwent plain radiographs and CT scan preoperatively and immediately postoperatively and were followed over time with systematic clinical and radiographic controls at 1, 3, 6, 12, and 24 months after surgery.

## 3. Results

The average surgical time was 113 minutes (range 35 to 240 minutes), and it was directly related to the number of screws implanted: the average time, reduced to 106 minutes using 4 pedicle screws, becomes 144 minutes with 6 screws and 171 minutes with 8 screws. Blood losses were not assessable intraoperatively. Postoperative analgesia was performed in all cases with a 36-hour lasting elastomeric pump containing an opioid and an NSAID. All monotrauma patients recovered the standing position in the second postoperative day on the average and were discharged on the fifth day. In polytrauma patients has been granted an immediate mobilization in the bed. 

The mean followup was 38 months, with a minimum of 6 months and a maximum of 72 months. 

All the cases, except one, have been considered healed after a 6-month control. Radiological examinations confirmed good spontaneous reconstruction of the anterior and posterior columns. Radiographic evaluation was performed through the measurement of the segmental kyphosis and the wedging deformity of the involved vertebral body [6]. Back pain, evaluated by VAS scale was 1.9 points at FU. Clinical evaluation was performed by subjective evaluation of the final results by patients themselves, and every patient was satisfied of surgical procedure. 

Radiographic evaluation showed a real improvement in the postoperative period (segmental kyphosis: 4.1 preop, −2.2 postop, and 2.7 FU kyphosis of the fractured vertebral segment: 12.2 preop, 5.9 postop, and 8.7 FU), but also a worsening of the segmentary kyphosis in the cases treated with CD Horizon Longitude (6.4 preop, 3.5 postop, and 7.8 FU) if implanted with multiaxial screws. (5.7 preop, 4.8 postop, 9.9 FU) (Table 2).

In two patients, one screw was found medial into the spine canal on the postoperative TC, without any clinical consequence.

**Figure 3:**
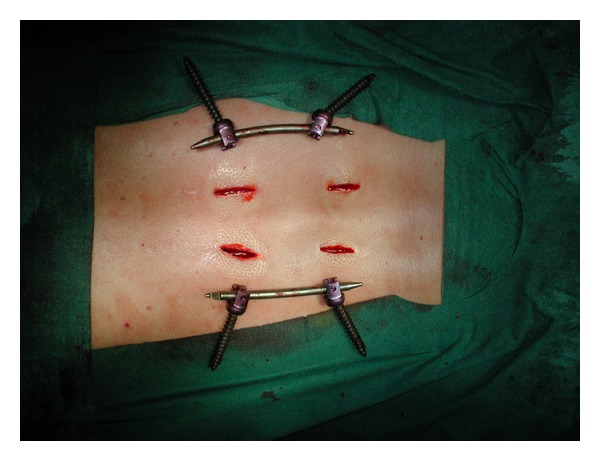
Percutaneous minimal invasive removal of the instrumentation.

At the beginning of our experience, we planned to remove all implants including L2 or a lower vertebra, no implant above T10 and all the implants in the thoracolumbar junction showing clinical (local pain) or mechanical problems (hardware failure or screws mobilization). We planned hardware removal in the lumbar spine as we were afraid that posterior fixation without fusion in such a mobile part of the spine could lead to hardware failure and consequently to clinical problems. Overall, the instrumentation has been removed in 23 patients (19%), in 5 cases due to a local complication and in 17 cases, as scheduled, because of implantationin the lumbar spine (Figure 3). The average delay from first surgery to implant removal was 9,5 months (range: 6–36). In the 17 patients in which implant removal had been planned, only 3 showed screws mobilization, and only 2 had pain. None of them showed pain or loss of sagittal alignment at six-month followup. 

## 4. Complications

The complications were divided according to a temporal order of appearance in intraoperative and postoperative. The latter were divided into early if they appear within one month from the date of surgery and late when they occurred after that period [7]. 

Depending on the severity, we divided complications into major and minor [8]. Major complications were those involving an increased hospitalization, or a second operation not scheduled. 

We recorded 12 complications (9.8%) divided into 4 intraoperative (3.3%), 6 early postoperative (4.9%), 2 late postoperative (1.6%). Four complications were minor (3.3%) and 8 major (6.5%). 

Intraoperative complications were all minor, related to mechanical instruments, which lengthened the surgical time but without any consequence for the patients. Early postoperative complications were all major: 4 mechanical, 1 neurological and 1 infectious complication. In 2 patients the screw head disconnected from the stem in the first postoperative day.

In one case, the patient was reoperated, while the other had to wear a brace for 3 months postoperatively. 

In 2 patients we recorded a pullout of the pedicle screws, 15 days and 20 days after surgery respectively. 

The first case was a 63-year old patient with 2 noncontiguous type A1 fractures (T11 and L1) undergoing MIS from T10 – L3 with bilateral pedicle screws in L1. The second case was a patient of 67 years fixed from T12 to L2 for a type A3 L1 fracture. In both cases, we performed the implant removal and a percutaneous augmentation of the vertebral bodies with cement.

The neurologic complication was a cauda equina syndrome which appeared in the second postoperative day in a patient treated for a type A L1 fracture by T12–L2 MIS. The patient underwent urgent surgical revision. In that occasion, we found an organized intradural hematoma sleeve enveloping the conus medullaris. We performed a complete removal of the hematoma with a microsurgical technique without finding the source of bleeding. Surprisingly no screw was found in the spinal canal during the revision surgery. The patient was subsequently sent to a rehabilitation center, and he completely regained the neurological functions in 2 months.

A 35-year old patient had a *Staphylococcus epidermidis* infection with surgical wound dehiscence. The patient had been submitted to MIS for a type A2 T11 fracture. Two and a half after surgery underwent surgical debridement and removal of the instrumentation resulting in healing of the infection. The patient wore a 3-point bodice for further 45 days, and the fracture healed with a residual kyphosis of 18 degrees.

Both late postoperative complications were major. In one case there was a nonunion in a patient with an A3 type T12 fracture, with initial kyphosis of 25°. Three months after surgery the patient still complained pain during weight bearing, and there was no evidence of healing on the CT scan. The patient underwent anterior fusion by thoracoscopic approach with incomplete pain relief. 

In the other case, there was an aseptic loosening of the screws in L5 in a young patient of 28 years, treated 3 years earlier by L3–L5 MIS for a B2 type L4 fracture. The patient had been scheduled for instrumentation removal 6 months after surgery, but he refused the operation. The patient underwent minimally invasive removal of fixation, with immediate disappearance of pain.

## 5. Discussion

The choice of treatment of the thoracic and lumbar spine injuries is related to many factors such as the type of fracture, the presence of neurological damage, associated injuries, patient's age, and others more. 

Conservative treatment of stable vertebral fractures is proposed with success by many authors [2, 3, 9–11], with different techniques: bed rest followed by external orthoses, extension gymnastics, plaster jacket in bed, or stand reduction [12]. Regardless of the methodology adopted, the treatment should be continued for a period of at least 3-4 months during which the patient care and cooperation is mandatory. The problems related to bed rest, particularly in the elderly, are countless, although difficult to calculate. Deep vein thrombosis may affect up to 30% of patients. Obesity, chronic obstructive pulmonary disease, venous incompetence, and psychiatric disorders are almost absolute contraindications to conservative treatment. 

In addition, today more and more patients need to return to their social and working life in a short time; therefore, surgery becomes the simplest way to shortcut recovery. In our experience, only 15% of the patients eligible for MIS opted for a conservative treatment. 

The rationale for applying MIS in the management of the spine fractures is to reduce the approach-related morbidity associated with the conventional technique: iatrogenic muscle denervation, increased intramuscular pressures, ischemia, pain, and functional impairment.

Because of the impossibility to perform a fusion, the minimally invasive percutaneous stabilization has been limited to relatively stable vertebral fractures, involving mainly bone component with a consistent possibility of spontaneous healing after immobilization; the screws and rods implanted acted as an internal fixator, leading to the biological healing of all fractures. Wang et al. comparing two groups of patients with thoracolumbar burst fractures, one treated by instrumented fusion, while the other just fixed without fusion, showed that there were no statistically significant differences in the long term between the two groups with a slight advantage, both for clinical than for radiographic parameters, for the group treated only with fixation without fusion [13]. This study further justifies the minimally invasive approach we have taken. 

PMMA injection through fenestrated cannulated screws provided additional stability in fixation procedures carried out on osteoporotic vertebral columns without affecting fracture healing.

Implant removal remains a controversial key point against this technique as it requires a second surgery and a general anesthesia, adding risks for the patient and costs for the hospital. Nevertheless, the real need for implant removal is probably much lower than that showed in our study as most of the patients who had the implant removed showed no clinical or radiological complications at the time of second surgery. Further studies are required to determinate the real need for hardware removal. The loss of correction, we observed during the followup for the cases treated with multiaxial screws could be explained by the possibility of this type of screws to have slight movement, also after implantation, between the head and the arm of the screw. For this reason, monoaxial screws should be considered for this kind of surgery, when it is possible. 

There are yet no studies that analyze the complications of MIS in thoracic and lumbar spine fractures. A retrospective study compares two groups of patients treated by MIS (10 patients) and arthrodesis with conventional technique (11 patients), with a minimum followup of 5 years. There is evidence of reduced blood loss for the group treated with MIS, but the study did not consider the complications occurred [14]. 

The complications in our series are comparable to those reported in the literature for conservative treatment, and much less than with open fusion.

## 6. Conclusion 

MIS in the treatment of thoracolumbar and lumbar spine fractures represents a good alternative option to conservative treatment.

Clinical and functional results are better or comparable, time of recovery is much quicker and the rate of complications is low. Implants need to be removed in case of complications or symptoms referred by the patient. Otherwise system hardware removal is mandatory only when fixation involves L2 or lower segments.

An adequate learning curve is important in order to minimize complications. The surgeon should also be confident about the instrumentation to reduce the duration of surgery and radiation exposure. The major complications primarily occur in the immediate postoperative period and can be related both to the implant and to the surgical procedure.

The correct surgical indication remains mandatory. Patients should be informed about the potential complications and the possible need for instrumentation removal.

## Figures and Tables

**Table 1:** Fractures distribution according to the type and level.

	A1	A2	A3	B1	B2	B3	C1	C2	TOT
T4								1	1
T5	3				1				4
T6	2	1							3
T7	2	1							3
T8	3			1	2			1	7
T9	3								3
T10	4				1				5
T11	6	3				1			10
T12	14	2	17		1				34
L1	15	4	27	1	1		1		49
L2	6	4	8	1					19
L3	3	1	5		1			1	11
L4	4	2	3		1				10
L5	2		2						4

TOT	67	18	62	3	8	1	1	3	163

**Table 2:** Radiographic evaluation.

	Segmental kyphosis			Vertebral wedging		
	Preop	Postop	FU	Preop	Postop	FU
Sextant + longitude	4.1	−2.2	2.7	12.2	5.9	8.7
Sextant	3.2	−4.8	0.3	11.8	4.5	8.2
Longitude	**6.4**	**3.5**	**7.8**	13.3	8.8	10
Long. polyaxial screws	**5.7**	**4.8**	**9.9**	14.3	9.3	10.3
Long. monoaxial screws	7.5	1	3.8	11.5	8	9.5
